# Managing Kienbock Disease’s Surgical Treatment and Outcome Analysis: A Case Report and Review of Literature

**DOI:** 10.7759/cureus.63352

**Published:** 2024-06-28

**Authors:** Ankit M Jaiswal, Sandeep Shrivastava, Yash Dhanwani, Rohan Chandanwale, Pallavi R Bhakaney

**Affiliations:** 1 Orthopedic Surgery, Datta Meghe Institute of Higher Education and Research, Wardha, IND; 2 Orthopedic Surgery, Government Medical College and Hospital, Akola, Akola, IND; 3 Physiotherapy, Dr. D. Y. Patil Vidyapeeth, Pune, IND

**Keywords:** kienbock’s disease, recent approaches, orthopedic approach, lichtman classification, scaphoid-capitate arthrodesis

## Abstract

A 38-year-old man without a severe traumatic history reported to the outpatient department (OPD) with wrist pain at the dorsal aspect, mild swelling, stiffness, and restricted mobility at the left wrist joint. The patient had been experiencing these symptoms for a year. There was sharp tenderness, graded as 4 above the lunate bone, on examination of the left wrist joint. Advanced imaging, which is magnetic resonance imaging (MRI), and radiographs suggested that the patient had Kienbock's illness. Typically, the surgical approach for Kienbock's used is wrist fusion or proximal row carpectomy. However, in this case, a novel strategy of bone grafting, scaphoid-capitate fusion, and lunate excision was adopted. This case report explains the outcome of our scaphoid-capitate arthrodesis, which was done to maintain functional mobility and relieve discomfort by halting the progression of carpal collapse and carpal-ulnar translation.

## Introduction

In 1910, Robert Kienbock, an Austrian radiologist, identified Kienbock's disease as a disorder marked by avascular necrosis of the lunate bone [1]. It is sometimes referred to as aseptic or ischemic necrosis of the lunate and osteonecrosis. This entity's pathophysiologic process is intricate [2]. The development of Kienbock's illness is attributed to a complex interplay of vascular and anatomic abnormalities, as well as various degrees of micro-trauma and insults. There is no one clear reason for it [3]. Depending on the stage at which the condition first manifests, patients with Kienbock disease often experience pain confined to the radiolunate facet, limited mobility, edema, and weakening in the affected hand. Lichtman and colleagues state that the illness can be divided into four stages using plain radiography [4]. This classification is the most clinically relevant since it aids in choosing the best course of therapy, making it incredibly dependable and reproducible [5]. After standard radiography, magnetic resonance imaging (MRI) is probably the next best diagnostic test. MRI is more sensitive and specific than bone scanning in the early stages of the disease, and it may assist in prognosis [6]. Thus, accurately illustrating the pathophysiology of such a rare disease requires early identification and appropriate healthcare management. In our case, we managed the case of Kienbock’s disease with single joint fusion, scaphocapitate fusion, which is a less invasive and joint motion-preserving technique as compared to the proximal row carpectomy and wrist fusion, which is generally preferred in the advanced grade of necrosis [7]. Comprehending these situations is crucial in advancing clinical expertise, optimizing therapeutic approaches, and enhancing prognoses for those with this illness.

## Case presentation

A 36-year-old male patient presented in the outpatient department (OPD) with the primary symptom of left wrist pain at the dorsal aspect for one year. Aggravating factors included the movement at the wrist joint and his daily routine activities. Since the patient is a shopkeeper by occupation, his major complaint was his inability to use his left hand to lift heavy objects at work. However, he reported no history of any trauma, tingling, or numbness. On clinical evaluation, localized minimal swelling over the wrist was present, and there was point tenderness over the wrist joint. The patient had painful and restricted flexion and extension movements at the wrist joint. Additionally, the radial artery was palpable, and all the sensations were intact in all the autonomous zones of the hand.

**Figure 1:**
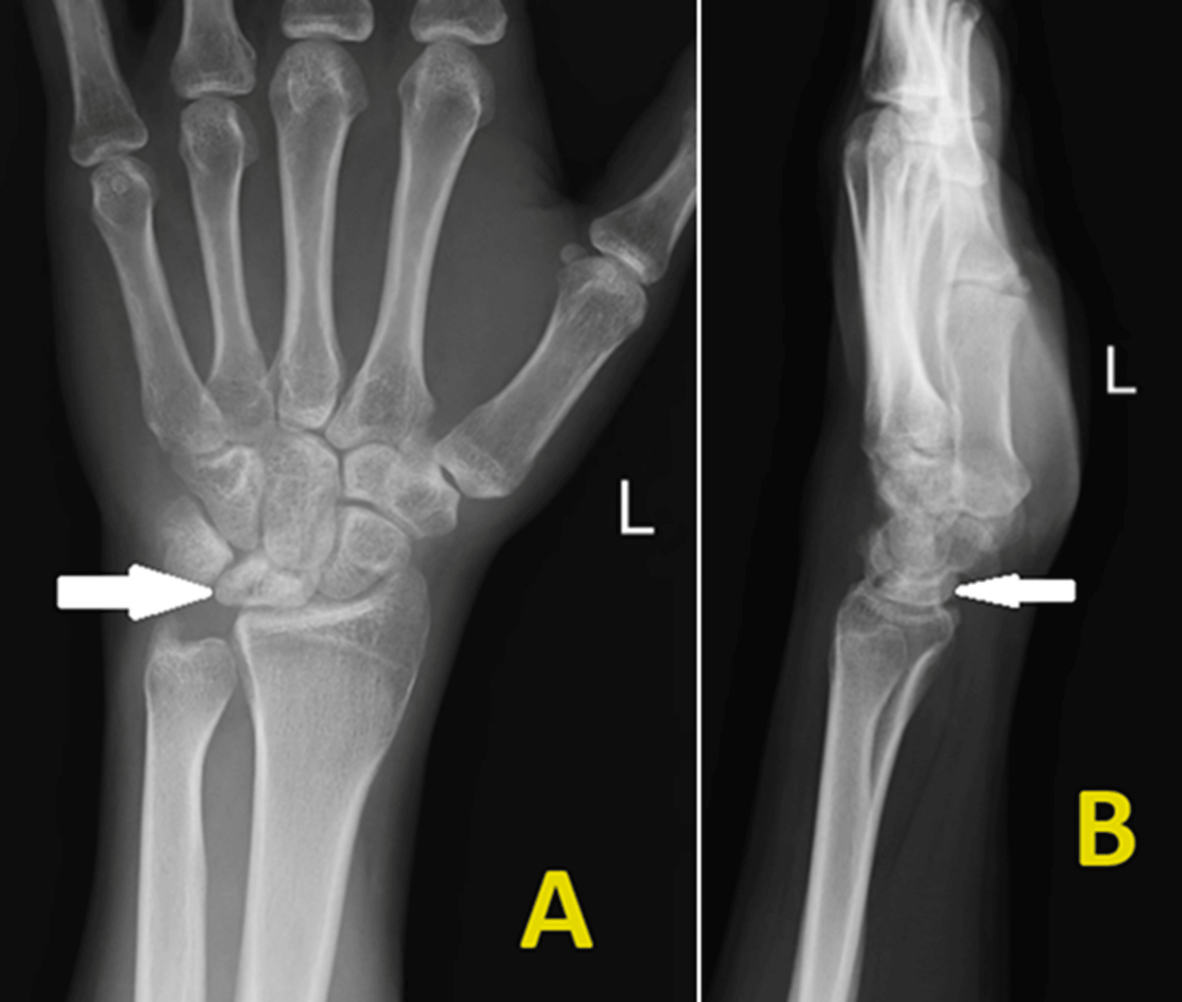
Preoperative radiograph of the left wrist showing sclerosis and fragmentation of the lunate bone, rotation of the scaphoid bone, and minimal flattening of the lunate. (A) Anteroposterior view and (B) lateral view.

An X-ray of the left wrist (anteroposterior and lateral view) was done to confirm the diagnosis. The imaging was suggestive of sclerosis of the lunate with a decrease in the height of the lunate and degenerative changes around the wrist joint (Figure 1).

**Figure 2:**
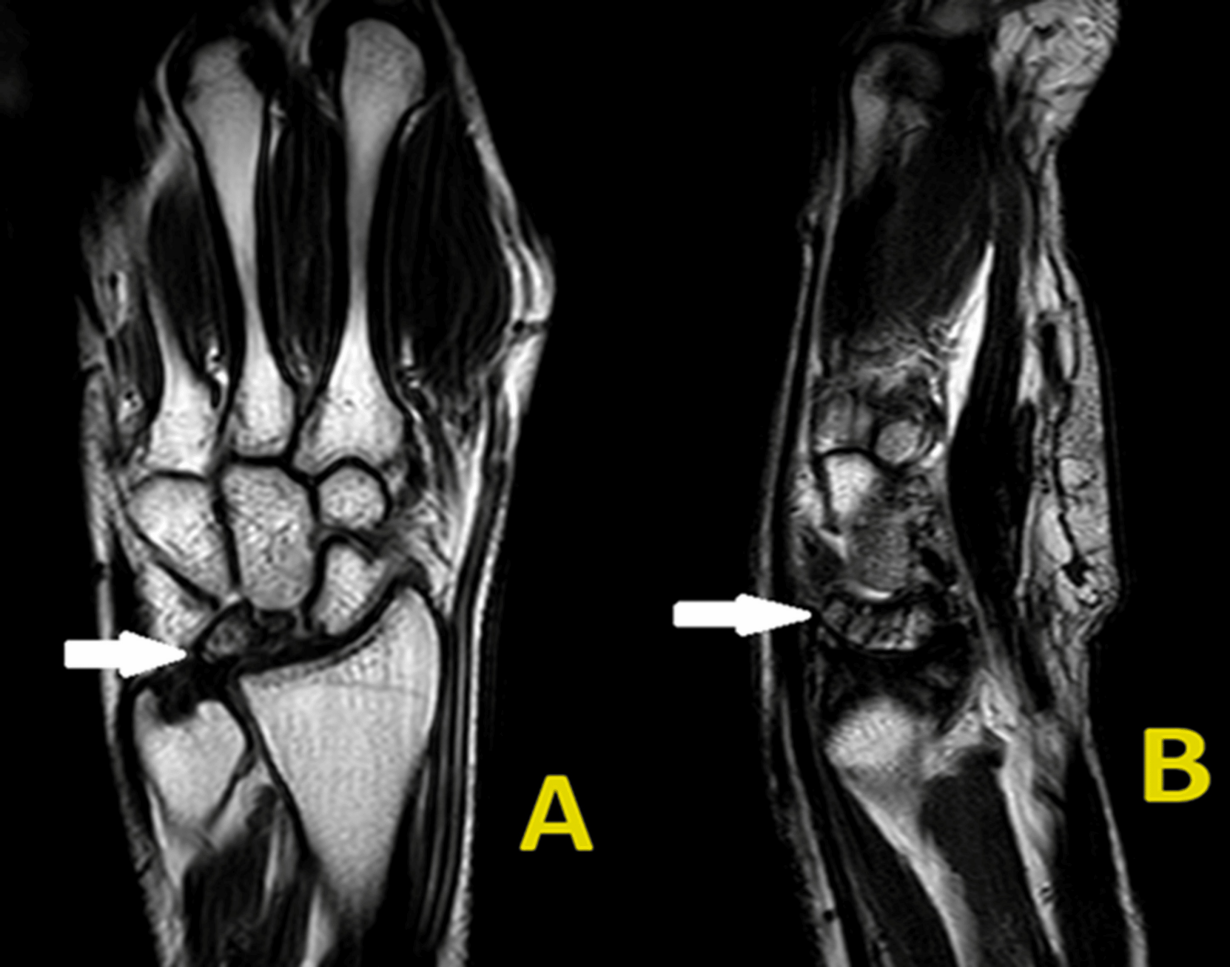
Preoperative MRI scan of the left wrist suggestive of sclerosis with marrow edema and collapse of the lunate bone with focal full-thickness chondral loss involving the articular surface of the distal radius and capitate suggestive of early secondary degenerative changes. (A) Anteroposterior view and (B) Lateral view. MRI: magnetic resonance imaging.

An additional MRI scan was also taken to get a better understanding of the condition (Figure 2).

A series of laboratory investigations were done, which included the following (Table 1).

**Table 1:** The laboratory investigations done pre-operatively. Hb: hemoglobin, gm: grams, TLC: total leukocyte count, cumm: cubic millimeter, mg/dl: milligrams per deciliter, nmol/l: nanomoles per liter, U/L: units per liter.

Investigations	Values	Normal range
Complete blood count	Hb: 13.2 gm%	Hb: 11-14 gm%
	TLC: 8500/cumm	TLC: 4000-11000/cumm
	Platelet count: 2,50,000/cumm	Platelet count: 1,50,000-4,00,000/cumm
Serum calcium	8.9 mg/dl	8.3-10.5 mg/dl
Vitamin D	45 nmol/l	50-120 nmol/l
Alkaline phosphate	250 U/L	130-260 U/L

**Figure 3:**
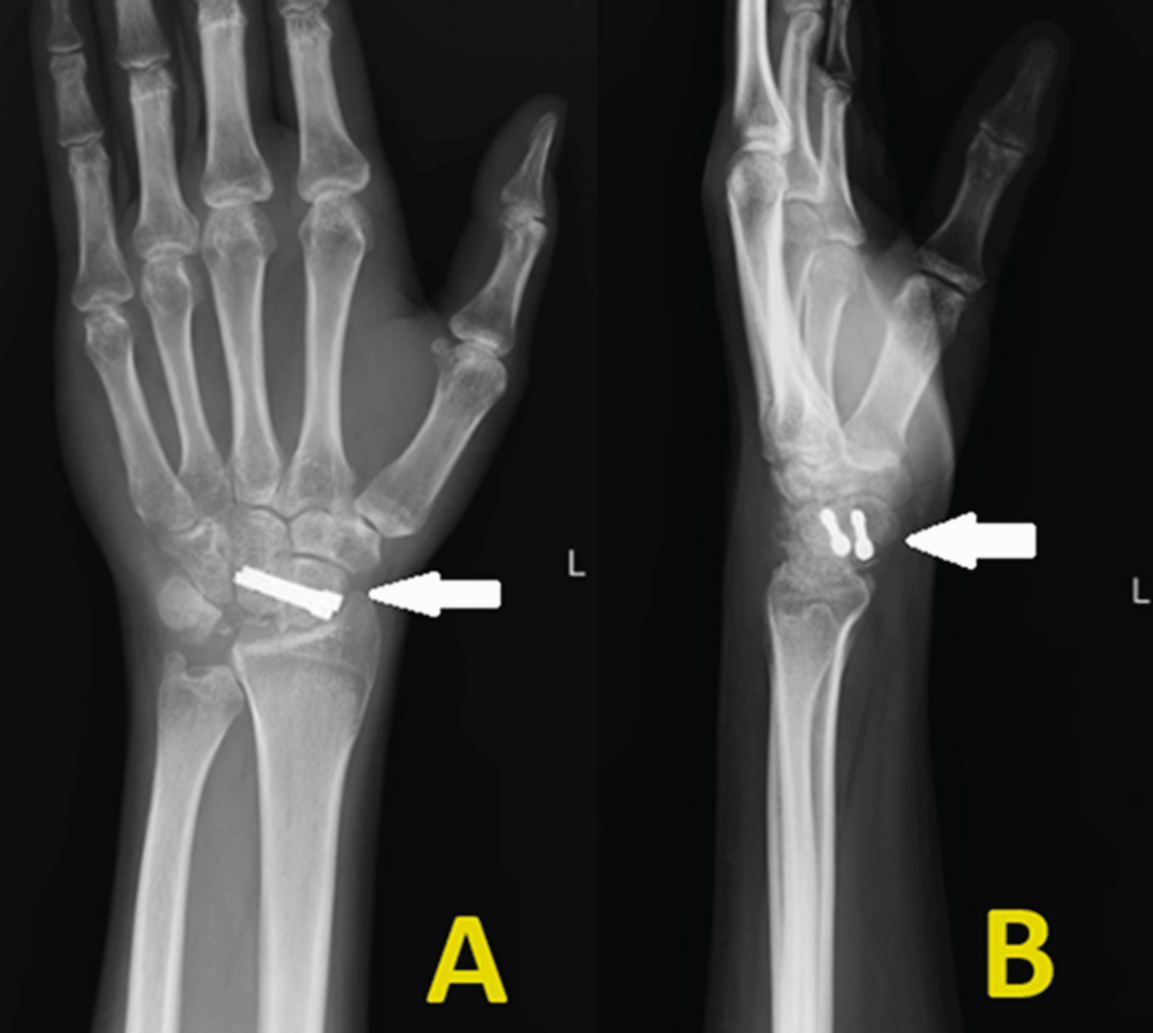
Postoperative radiograph of lunate excision and scaphocapitate arthrodesis. (A) Anteroposterior view and (B) Lateral view.

The patient underwent excision of the lunate with single joint fusion, i.e., scaphocapitate arthrodesis (SCA) through the dorsal approach to the wrist. Through the third and fourth extensor compartments, the lunate was excised and the scaphoid-capitate joint was denuded. Fusion was performed and fixed with a 2 Herbert screw and with a cancellous bone graft harvested from the ipsilateral radius. Immediate post-operative radiograph was found to be satisfactory (Figure 3). The patient was put in a below-elbow splint for immobilization for six weeks postoperatively. After six weeks, extensive physiotherapy rehabilitation was done, and the patient achieved a full range of motion (ROM) within three weeks with no further complications.

Follow-up and outcomes

**Figure 4:**
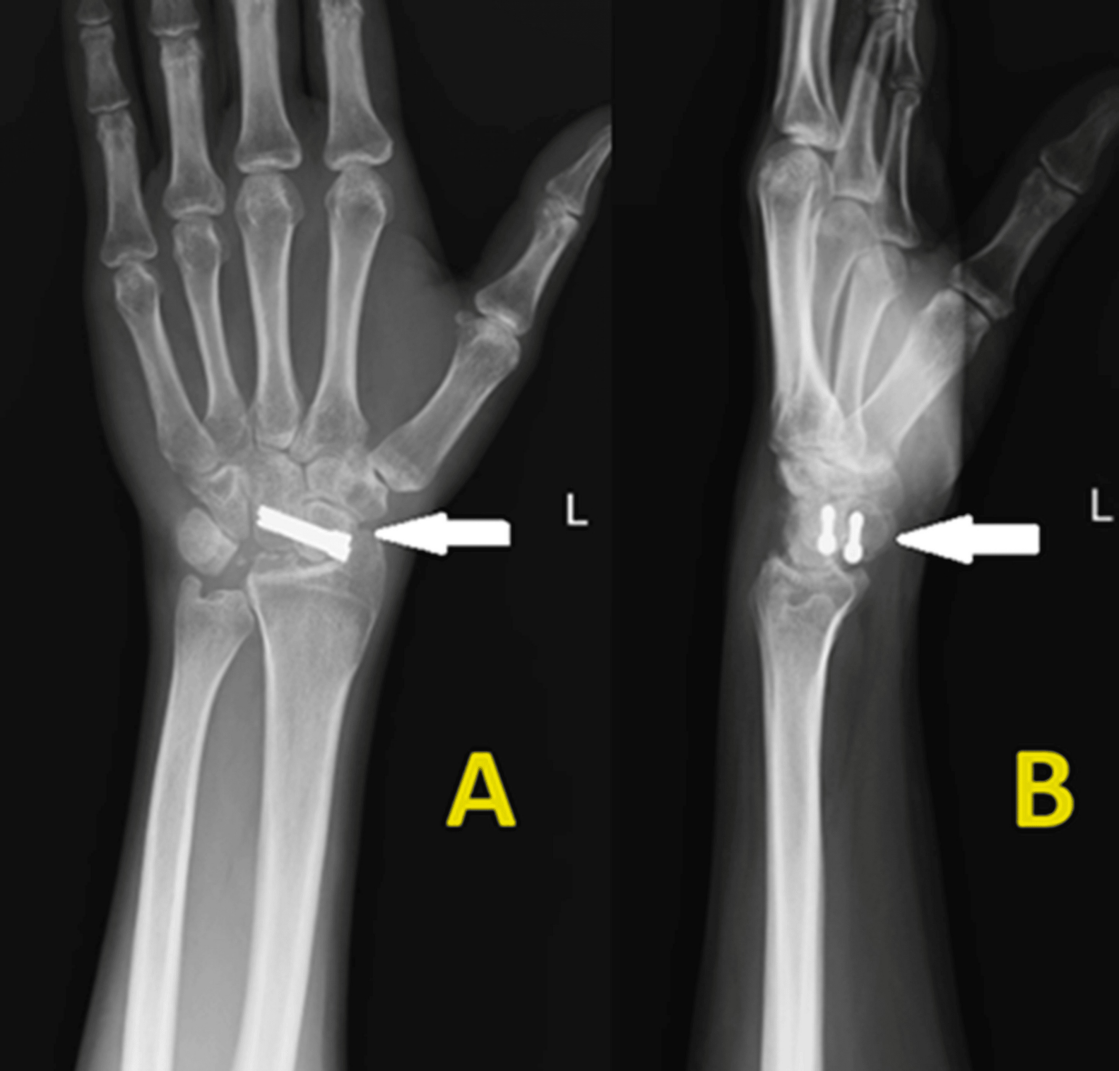
Postoperative radiograph after one-year follow-up of lunate excision and scaphocapitate arthrodesis. (A) Anteroposterior view and (B) Lateral view.

One-year follow-up radiographs (Figure 4) showed good scaphoid-capitate fusion with the screw in situ, and the radioscaphoid articulation did not show any degenerative changes. Functional evaluation was performed pre- and post-operatively at one-year follow-up.

## Discussion

Kienbock's illness continues to be a challenging clinical concern. If left untreated, it frequently progresses and causes joint destruction in three to five years. There are various choices for treatment, mostly determined by the stage of presentation. Options can vary, but generally, they can be divided into three main categories: treating carpal instability and collapse with salvage surgeries, revascularizing the lunate, or unloading the lunate. The advanced stage of Kienbock's illness, or stage 4, can be treated via wrist fusion, proximal row corpectomy, and limital carpal fusion, per Lichtman's classification. The justification for managing SC arthrodesis aligns with the widely accepted notion of scaphoid-trapezium-trapezoid (STT) arthrodesis. To retain functional motion and relieve discomfort, STT and SC arthrodesis both seek to halt the advancement of carpal collapse and carpal-ulnar translation. Scaphoid-capitate fusion, which only requires the fusion of one joint, is simpler than the STT fusion approach, which requires the union of two joints. In our case study, SCA was performed [8]. The study done by Watanabe et al. documented the presence of cartilage destruction arthroscopically in the radiolunate joint despite the absence of osteoarthritic abnormalities on the radiological assessment of grade III B Kienbock's illness; thus, it was concluded that the pathology can also occur in the capitulate joint [9]. After this study, it was noted that lunate excision and intercarpal arthrodesis, as suggested by Nakamura et al., were more appropriate because proximal row carpectomy was not shown to be entirely acceptable [10].

Later, Takase and Imakiire et al. showed that intercarpal arthrodesis in conjunction with lunate excision was an effective treatment for Kienbock's illness [11]. The goal of limited intercarpal arthrodesis (LCA) is to maintain carpal height and lessen the strain on the lunate by positioning the scaphoid bone appropriately during arthrodesis between the carpal bones. Scaphoid-trapezium-trapezoid (STT) arthrodesis and SCA can lessen the pressure on the lunate in this condition. However, there is no reduction in carpal loading even if another LCA technique is used; it only relieves the symptoms of Kienbock's illness [12]. Biomechanical investigations have demonstrated that in addition to capitate-hamate-arthrodesis (CHA), SCA and STT arthrodesis resulted in a noteworthy reduction in loading on the lunate [13]. In a recent biomechanical investigation, Li et al. reported that STT arthrodesis is significantly more valuable than SCA in preventing rotatory semi-dislocation of the scaphoid and preserving wrist stability [14]. In this instance, the patient had SCA after having their lunate removed, and their carpal height did not significantly change. One definition of SCA is a technique that yields results as successful as those of STT. The non-union rates are half with this method. This approach can be used arthroscopically in conjunction with other procedures and has been demonstrated to offer excellent treatment with low rates of non-union. However, patients undergoing long-term follow-up frequently showed radiographic indications of radioscaphoid arthritis [15]. In our case, an in-depth follow-up was required to identify this issue. When compared to the unaffected side, wrist flexion is reduced by 43-54% and wrist extension by 40-54% in SCA. It has been noted that the reduction in wrist joint range of motion is not as substantial as that seen with STT arthrodesis. It has been demonstrated that SCA increases grip strength. 75-151% of this rise has been documented [16]. Although grip strength and wrist joint range of motion were significantly increased in our case with LCA, they were not as high as those on the unaffected side. Several studies have revealed that given the high likelihood of complications combined with LCA, the wrist may advance to arthrodesis [17]. Revision surgery has been necessary on occasion in trials where a corticocancellous autograft with a screw or staple has been used in SCA because of non-union. Inadequate stability at the fusion site may be the primary cause of the variation in fusion rates. High rates of non-union in LCA with K-wire or screw fixation were observed in comparative investigations by Tambe et al. [18]. The incidence of non-union complications was reduced to almost non-existent when the fusion was rigidly fixed by grafting. In our instance, fixation was attained by the Herbert screw, resulting in a successful arthrodesis.

## Conclusions

The SCA, or limited carpal fusion, can be considered an option in grade 4 Lichtman osteonecrosis of the lunate. Limited carpal fusion with a bone graft leads to minimal tissue trauma and good postoperative ROM. We report a good functional outcome in our case with limited carpal fusion, i.e., scaphocapitate arthrodesis and lunate excision.
